# Elementary Physiology for Students

**Published:** 1893-07

**Authors:** 


### Book Reviews.

ELEMENTARY PHYSIOLOGY FOR STUDENTS, by Alfred T. Schofield, M. D., M. R. C. S., late House Physician to the London Hospital ; Special Lecturer National Health Society. In one handsome 12mo. volume of 385 pages, with 227 engravings and 2 colored plates. Cloth, $2. Philadelphia, Lea Bros. & Co.

We hope this work may supplant the "quiz compends" on Physiology. It does not aim to take the place of works like Landois', but is a compact text-book, designed especially for students. The author has avoided useless discussions of various theories, and deals with the established facts of physiology. The book is very fully illustrated with both engravings and colored plates, is of a convenient size and reasonable price. We heartily commend it to the medical student.

